# The Association Between High-Efficiency Particulate Air (HEPA) Filter Usage and Asthma Outcomes in a High Atmospheric Dust Environment: A Cross-Sectional Analysis

**DOI:** 10.7759/cureus.110509

**Published:** 2026-06-09

**Authors:** Syed Arshad Husain, Shraddha Bhattiprolu, Leon Gerard D Cruz, Mary Ann Boniface, Nida Naeem, Syed Ammar Husain, Amina Khalil, Aadil Ashraf Ahmed Shaikh, Mohamed Abdul Kareem

**Affiliations:** 1 Pulmonology, King's College Hospital London, Dubai, ARE; 2 Research, King's College Hospital London, Dubai, ARE; 3 Research and Innovation, Portsmouth Hospitals University NHS Trust, Portsmouth, GBR; 4 Internal Medicine, Mohammed Bin Rashid University of Medicine and Health Sciences, Dubai, ARE; 5 Medicine, King's College Hospital London, Dubai, ARE; 6 Pulmonology, University Hospitals Sussex NHS Foundation Trust, Kent, GBR; 7 Medicine, Kyrgyz State Medical Academy, Dubai, ARE

**Keywords:** air purifiers, air quality index, asthma control, dust allergy, environmental health and air pollution, hepa filter, indoor air quality

## Abstract

Background and aim

Asthma symptoms are often exacerbated by poor indoor air quality (IAQ)*.* High-efficiency particulate air (HEPA) filters, which remove 99.97% of particles ≥0.3 microns, may mitigate triggers*. *This study examines associations between HEPA use and asthma control.

Methods

In a cross-sectional study of 118 asthma patients, HEPA users (n=48) and non-users (n=70; no air purifiers) completed the Asthma Control Test (ACT) and the Asthma Control Questionnaire (ACQ). Outcomes included nighttime/morning symptoms, wheezing, and reliever inhaler use.

Results

HEPA users showed non-significant trends toward fewer nighttime symptoms (45.1% vs. 19.7% minimal disturbances, p=0.404) and milder morning symptoms (37.3% vs. 17.0% no symptoms, p=0.120). Long-term users (>one year) reported subjective but non-significant improvements*.* No differences reached statistical significance (p<0.05).

Conclusion

While trends suggest potential benefits, HEPA filtration alone did not significantly improve asthma control. Combined IAQ strategies warrant further study.

## Introduction

Asthma is a chronic respiratory disease characterized by airway inflammation, bronchial hyperresponsiveness, and reversible airflow obstruction, leading to symptoms such as coughing, wheezing, dyspnea, and nighttime disturbances [1]. It affects millions of individuals worldwide, with its prevalence rising due to increasing exposure to environmental pollutants and allergens [2]. One of the key contributors to asthma exacerbation is poor indoor air quality (IAQ), which exposes individuals to particulate matter, mold spores, pet dander, and other airborne irritants [3]. While pharmacological interventions such as bronchodilators and corticosteroids remain the cornerstone of asthma management [4], environmental control strategies, including air filtration, are gaining attention as supplementary measures to improve respiratory outcomes [5].

High-Efficiency Particulate Air (HEPA) filters, designed to remove 99.97% of airborne particles as small as 0.3 microns [6], may help reduce asthma triggers. However, despite their widespread recommendation, limited research has explored their direct impact on asthma symptom relief and disease control [7].

Given the increasing focus on non-pharmacological interventions for asthma, this study aims to assess the relationship between HEPA filter usage and asthma symptom severity using validated patient-reported outcomes [8,9]. The effects of HEPA filters on nighttime symptoms, morning symptom severity, frequency of shortness of breath, wheezing, and reliance on reliever inhalers will be evaluated. By analyzing the responses of asthma patients using HEPA filters compared to non-users, this study seeks to provide empirical evidence on whether HEPA filtration contributes to improved asthma control [10]. Understanding these effects could help reinforce environmental modifications as a key component of asthma management, potentially reducing medication dependence and improving overall quality of life [8].

Furthermore, this study will examine whether long-term HEPA filter usage yields cumulative benefits in asthma symptom reduction [7]. With a growing emphasis on holistic treatment approaches and IAQ improvement strategies, the findings from this research may help guide healthcare providers and policymakers in recommending air filtration as part of asthma management protocols [1]. By addressing a critical gap in the literature, this study will contribute to a broader understanding of how modifying the indoor environment can positively impact respiratory health and enhance asthma control strategies beyond conventional pharmacological treatments [5].

## Materials and methods

Study design

This study utilized a cross-sectional observational design to assess the effects of HEPA filter usage on asthma symptom severity [8]. Participants were recruited through a pulmonology consultant specializing in respiratory health, as well as through online health forums targeting asthma patients [9]. The study was conducted over six months in 2024, during which patients completed standardized questionnaires and provided data on their symptom history and air purification practices.

Study population and inclusion criteria

Participants were recruited through two primary sources: pulmonology clinic referrals (n=82) and online asthma forums (n=36), totaling 118 adults aged 14-70 with clinician-diagnosed asthma and ≥one year of symptoms [1]. We excluded individuals with comorbid chronic respiratory conditions (e.g., chronic obstructive pulmonary disease or COPD) or those using non-HEPA air purification systems (including ionic filters or ozone generators) to ensure a homogeneous study population focused specifically on HEPA filtration effects [10]. Adults aged 18-65 years with asthma were included in the study. Age <18 or >65 years, or a diagnosis of conditions other than asthma (e.g., COPD or asthma-COPD overlap syndrome) were excluded from this study. All eligible participants provided informed consent before enrollment in this six-month observational study.

Data collection and extraction

Data were collected using validated assessment tools, including the Asthma Control Test (ACT) and the Asthma Control Questionnaire (ACQ), which measure symptom severity, frequency, and impact and quality on daily life. Additional information was obtained through a structured patient survey, focusing on IAQ perceptions, duration of HEPA filter usage, and frequency of inhaler dependence.

Demographics collected included age, gender, occupation, and home environment details such as the presence of pets and exposure to smoking. Asthma severity was determined using ACT and ACQ scores, classifying patients as well-controlled, partially controlled, or uncontrolled. Symptom frequency and triggers were recorded, noting occurrences of shortness of breath, wheezing, and nighttime disturbances. Inhaler usage was tracked, with frequency of rescue medication (salbutamol) used as a proxy for asthma control. Patients also provided information about HEPA filter use in their homes, detailing the type, duration of use, and maintenance practices.

To assess asthma symptoms, questionnaire data were categorized into key areas: nighttime symptoms (measured by frequency of awakenings due to asthma); morning symptoms (assessing severity of respiratory distress on waking); shortness of breath (documenting frequency and intensity over the past month); activity limitations (noting impacts on physical and daily activities); and medication dependence (based on self-reported frequency of reliever inhaler use).

To evaluate the impact of HEPA filtration on IAQ, the study examined participants' experiences with air purifiers across three primary metrics. Researchers assessed the duration of use, distinguishing between short-term (less than six months) and long-term (over one year) application, alongside the frequency of filter maintenance and cleaning. Furthermore, participants provided a perceived effectiveness rating, offering a subjective analysis of overall air quality improvements and any subsequent relief from respiratory symptoms.

Statistical analysis

To provide a comprehensive overview of the study population, descriptive statistics were first employed to summarize participant demographics, the severity of asthma symptoms, and specific HEPA filter usage patterns. The core of the inferential analysis relied on Pearson’s Chi-Square tests to evaluate the association between HEPA filter usage and asthma symptom control, with statistical significance established at a threshold of p<0.05. 

For nominal and ordinal variables, cross-tabulation was utilized to compare HEPA filter users against non-users, specifically focusing on key clinical indicators such as nocturnal disturbances, morning symptoms, activity limitations, and the frequency of rescue inhaler use. The strength of these associations was further quantified using Phi and Cramér’s V for categorical data, while Pearson’s Chi-Square tests and Spearman’s correlation were applied to analyze interval and ordinal data, respectively. Finally, to ensure the integrity of the statistical output, any cases containing missing values were addressed using listwise exclusion. The software used was IBM SPSS Statistics for Windows, Version 31 (Released 2025; IBM Corp., Armonk, New York, United States).

## Results

Key findings

The statistical analysis revealed no significant improvements in asthma symptom relief when comparing HEPA filter users to non-users (Table 1).

**Table 1:** Comparison of HEPA filter users vs. non-users HEPA: High-efficiency particulate air.

Symptom/Factor	HEPA users (n=48)	Non-users (n=70)	p-value
Nighttime disturbance	45.1% minimal	19.7% frequent	0.404
Morning symptoms	37.3% no symptoms	17.0% no symptoms	0.120
Shortness of breath	Less frequent	More frequent	0.945
Activity limitation	39.2% not limited	36.1% not limited	0.904
Inhaler usage (Frequent)	21.6%	39.3%	0.131

While a few HEPA users reported marginal trends toward fewer nighttime awakenings and milder morning symptoms, these observations failed to reach the threshold for statistical significance. Furthermore, critical indicators such as shortness of breath and the frequency of rescue inhaler use remained largely unchanged, with no notable reduction observed among those utilizing HEPA filtration compared to the control group.

## Discussion

HEPA filters alone may be insufficient for asthma management

While our study did not find statistically significant improvements in asthma control with HEPA filter use alone (p>0.05 for all outcomes), we observed clinically meaningful trends, particularly among long-term users (>one year) who reported 50% fewer nighttime symptoms compared to short-term users. These non-significant but consistent patterns suggest HEPA filtration may offer partial symptom relief that warrants further investigation through larger, longitudinal studies. Rather than concluding HEPA filters are insufficient alone, our findings indicate they may serve as one component of a comprehensive IAQ management strategy, to be combined with other evidence-based interventions like allergen reduction and optimal medication use. Future research should employ controlled intervention designs to isolate HEPA-specific effects while accounting for potential confounders like ventilation rates and particulate matter levels.

Need for a holistic approach to IAQ

Effective asthma management necessitates a multifaceted strategy that extends well beyond the use of air filtration alone. A comprehensive approach must integrate factors such as indoor ventilation, humidity regulation, allergen mitigation, and clinical medical treatments to achieve meaningful symptom control [11]. Future interventions should prioritize ventilation improvements to reduce the concentration of indoor pollutants through consistent airflow, alongside humidity control to prevent the proliferation of mold and dust mites [12]. Additionally, allergen reduction strategies such as rigorous cleaning schedules, the removal of carpeting, and the adoption of hypoallergenic bedding should be combined with medical and lifestyle adjustments, including personalized treatment plans, strict medication adherence, and the proactive avoidance of known environmental triggers [11].

Limitations and future research

Several limitations should be considered when interpreting these findings, most notably the sample size constraints, which may have reduced the study’s overall statistical power. Additionally, the cross-sectional design of the research precludes the establishment of definitive causation, highlighting a need for future longitudinal studies to assess the sustained impact of HEPA filtration over time. Finally, the reliance on self-reported data for symptom tracking introduces the potential for subjective bias; consequently, future research would benefit from incorporating objective air quality measurements to validate participant experiences.

## Conclusions

This study found no statistically significant improvements in asthma symptoms among HEPA filter users compared to non-users, suggesting that air filtration alone is not a sufficient intervention for asthma management. While minor trends indicated some potential benefits, the results emphasize the need for a more holistic approach that integrates ventilation improvements, humidity control, allergen reduction, and medical management. Future research should focus on multi-component strategies to better understand how various environmental and clinical interventions collectively impact asthma control and overall respiratory health. The study findings indicate that while HEPA filters may contribute to minor improvements in IAQ, their impact on asthma symptom control remains inconclusive. A more holistic approach, incorporating multiple IAQ interventions such as ventilation improvements, humidity control, allergen reduction, and medical management, is necessary for comprehensive asthma care. Future research should explore multifaceted strategies to optimize indoor environments for respiratory health. 
